# Failure Mechanisms of Hollow Fiber Supported Ionic Liquid Membranes

**DOI:** 10.3390/membranes6020021

**Published:** 2016-03-23

**Authors:** Matthew Zeh, Shan Wickramanayake, David Hopkinson

**Affiliations:** 1 National Energy Technology Laboratory, 3610 Collins Ferry Road, Morgantown, WV 26507, USA; mzeh@utexas.edu; 2 AECOM Technology Corporation, 626 Cochrans Mill Road, Pittsburgh, PA 15236, USA; wickramw@netl.doe.gov

**Keywords:** supported ionic liquid membrane, hollow fiber, stability, failure mode, bubble point, rupture, collapse

## Abstract

Hollow fiber supported ionic liquid membranes (SILMs) were tested using the bubble point method to investigate potential failure modes, including the maximum transmembrane pressure before loss of the ionic liquid from the support. Porous hollow fiber supports were fabricated with different pore morphologies using Matrimid^®^ and Torlon^®^ as the polymeric material and 1-hexyl-3-methylimidalzolium bis(trifluoromethylsulfonyl)imide ([C_6_mim][Tf_2_N]) as the ionic liquid (IL) component. Hollow fiber SILMs were tested for their maximum pressure before failure, with pressure applied either from the bore side or shell side. It was found that the membranes exhibited one or more of three different modes of failure when pressurized: liquid loss (occurring at the bubble point), rupture, and collapse.

## 1. Introduction

Supported ionic liquid membranes (SILMs) remain underutilized because of the uncertainty of their mechanical stability under pressure, despite their potential use in gas separation applications due to their high permeabilities and selectivities [1,2,3]. An SILM is a composite structure consisting of a porous support saturated with an ionic liquid (IL). ILs are salts that are liquid at room temperature that have negligible vapor pressure. The IL is suspended in the pores of the support by capillary forces alone, which makes the design of the support critical to achieving adequate stability. Recently some groups have begun testing the gas permeance properties of SILMs in a hollow fiber geometry [4,5,6,7], which have the practical benefit of a higher surface area to volume ratio than flat sheet membranes. In this configuration, there remain questions regarding the stability of the IL component, plasticization of the polymeric support, and the general mechanical stability of the fiber support.

In practice, if the trans-membrane pressure exceeds the capillary pressure, the suspended IL will be displaced from the pores of the fiber, leading to failure of the membrane. One technique to determine the maximum allowable trans-membrane pressure is the bubble point method, in which gas pressure is slowly increased to one side of the membrane until it begins to displace liquid from the pores and convectively flow through the membrane (the bubble point) [8]. In a previous study, the bubble point method was used to characterize SILMs utilizing the ILs 1-hexyl-3-methylimidalzolium bis(trifluoromethylsulfonyl)imide ([C_6_mim][Tf_2_N]) and 1-ethyl-3-methylimidazolium ethyl sulfate ([C_4_mim][EtSO_4_]) on flat sheet supports of various materials, pore morphologies, and pore sizes [9]. It was shown that the Laplace-Young equation can be used to predict the bubble point of the SILMs after determining an effective maximum pore size for the support using a known calibration liquid. Further, it was found that changing the liquid caused the bubble point to vary in proportion to the surface tension of the liquid, but not by the contact angle. This conclusion was in agreement with several other literature reports indicating that the contact angle of the Laplace-Young equation should always be considered to be zero for this application [10,11].

The long-term stability of flat-sheet SILMs under low trans-membrane pressure has been studied previously. Cserjesi and co-authors studied SILMs using the IL 1-{2-[2-(etoxy)-etoxy]-ethyl}-3-methylimidazolium hexafluorophosphate with a polyvinylidene fluoride (PVDF) support material, which were tested at 25 °C and a trans-membrane pressure of 1.2 bar [12]. These SILMs showed an increase in CO_2_/H_2_ selectivity over the course of eight separate, hour-long cycles for which the SILM was held at constant pressure for each individual cycle. Hanioka and co-authors demonstrated the stability of an SILM using the IL N-aminopropyl-3-methylimidazolium bis(trifluoromethylsulfonyl)imide ([C_3_NH_2_mim][Tf_2_N]) using a CO_2_/CH_4_ feed gas with a feed pressure of 1 bar [13]. The selectivity exhibited by the SILM remained almost constant over a period of 260 days. Scovazzo and co-authors tested the long-term stability of hydrophilic-PVDF and hydrophilic-polyethersulfone (PES) membranes with imidazolium-based ILs using CO_2_/CH_4_ and CO_2_/N_2_ mixed gas feeds [14]. The authors reported no degradation in performance of the SILMs under continuous operation from 24 to 106 days pressurized at a feed pressure of 2–4 bar. While these studies provide valuable insight into the long term stability of membranes in low pressure regimes, the maximum operating pressure of SILMs, particularly using hollow fiber supports, requires further investigation.

To date, studies of the mechanical properties of hollow fibers have been focused on neat polymeric materials. Tasselli and Drioli demonstrated polymeric micro-/ultra-filtration selective membranes of poly(ether ether ketone) with Young’s Moduli ranging from 65 to 90 MPa and tensile strengths of 30–40 bar through the variation of bore fluids in preparation of hollow fibers using the dry-wet spinning method [15]. In a study by Zhang and Lu, PVDF hollow fiber membranes prepared for hemodialysis showed a relationship between total porosity and tensile strength [16]. This study found that an increase in porosity causes a decrease in the tensile strength of the fiber. When saturated with water, it was also found that the bubble point of these hollow fibers decreased with increasing maximum pore size. Sukitpaneenit and Chung also noted a decrease in tensile strength and Young’s modulus with increasing porosity for PVDF hollow fibers [17].

The gas transport properties and tensile strength of SILMs using Matrimid^®^ and Torlon^®^ hollow fiber supports with the IL [C_6_mim][Tf_2_N] have been studied previously [5]. The study found that increasing the porosity of the support leads to an increase in both the CO_2_ permeance and the CO_2_/H_2_ selectivity of the membrane due to an increase in the IL content. Further, membranes using Matrimid^®^ and Torlon^®^ supports showed approximately the same CO_2_ permeance and CO_2_/H_2_ selectivity regardless of the support material when of a similar porosity. This was due to a relatively small co-permeance contribution of the polymer when compared with the IL [6]. Although an increase in porosity benefits the gas transport properties of a supported ionic liquid membrane, it negatively affects the mechanical stability of the hollow fiber. Finally, Torlon^®^ fibers had more robust mechanical properties than Matrimid^®^ fibers with a similar porosity, although Torlon^®^ has a lower glass transition temperature. Molecular simulations showed that Torlon^®^ polymer chains have interlocking behavior that results in high strength and a resistance to ionic liquid plasticization effects [5].

In this paper, the maximum operating pressure of supported ionic liquid hollow fiber membranes was studied using the bubble point method. Two polymer support materials, Matrimid^®^ and Torlon^®^, were tested each using a low porosity and a high porosity conformation. Hollow fibers were tested dry and saturated with the IL [C_6_mim][Tf_2_N]. Pressure was applied to either the bore or shell side of each hollow fiber configuration to determine the maximum pressure before failure as well as the failure mode.

## 2. Results and Discussion

### 2.1. Hollow Fiber Morphology

Four different hollow fiber supports were fabricated using Matrimid^®^ and Torlon^®^ polymers at room temperature with the dope compositions shown in Table 1. Composition *a* and *b* used Matrimid^®^, while fibers *c* and *d* were made from Torlon^®^. In order to increase the hollow fiber porosity in Matrimid^®^ and Torlon^®^ composition *b* and *d*, respectively, lower polymer concentrations were used than in compositions *a* and *c*. In all compositions, LiNO_3_ was used as an additive to form a complex with NMP in the polymer dope, increasing the number of pore spaces after solvent extraction [18].

Matrimid^®^ and Torlon^®^ were chosen for the fabrication of polymeric hollow fibers due to their high glass transition temperatures (330 and 270 °C, respectively), high Young’s modulus, and high tensile strength, making them very stable materials for membrane supports [19,20,21,22]. Both are glassy polymers with mid-level selectivity for light gases and low permeability.

**Figure 1:**
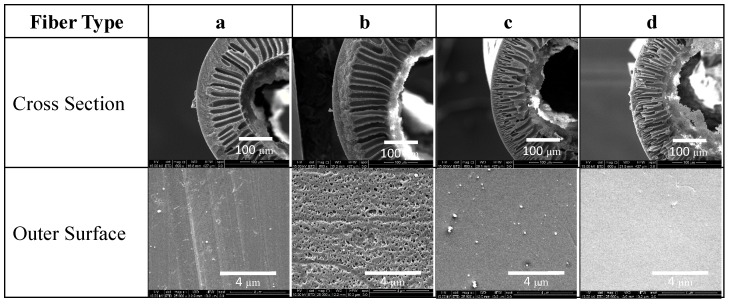
SEM images of the cross section (top) and outer surface (bottom) of the four hollow fiber membrane supports: (**a**) low porosity Matrimid^®^; (**b**) high porosity Matrimid^®^; (**c**) low porosity Torlon^®^; (**d**) high porosity Torlon^®^.

Through SEM image analysis the fiber dimensions, including outside diameter (*OD*), inside diameter (*ID*), and bulk porosity (*v_bulk_*), were determined for each fiber type (Figure 1, Table 2). Hollow fibers consisted of a series of finger-like macro-voids extending from the inner surface to the middle region of the cross section, and a meso-porous region located between the outer surface and macro-voids. Although the general structure of the hollow fibers was similar, Matrimid^®^ fibers tend to have a thicker meso-porous region (~40 μm), while Torlon^®^ fibers have a smaller meso-porous region thickness (~2 μm) with macro voids that extend through nearly the entire cross section of the fiber. For the SILMs presented here, this results in a thicker selective layer for Matrimid^®^ hollow fibers since IL can be easily flushed out from the macro-voids and retained in the meso-porous region [5,6]. Matrimid^®^ fiber *b* has a highly porous outer surface with sub-micron sized pores, while all the pores on the outer surface of all other fibers could not be clearly resolved from the SEM images shown. More porous supports, such as fiber *b* will result in SILMs with higher gas transport performance due to an increased ionic liquid content when the pores are fully saturated. However, larger pores also have less capillary resistance for retaining liquids under pressure, as is known from the Laplace-Young equation [23]:

(1) $$\Delta p_{c}=4\gamma \cos\theta /d_{p},$$

where Δ*p_c_* is the critical trans-membrane pressure (the bubble point), γ is the surface tension of the liquid-gaseous interface, θ is the contact angle at the liquid and solid surface interface, and *d_p_* is the largest pore diameter. Since the hollow fibers in this study are asymmetric and generally consist of large pores near the bore that taper into smaller pores at the shell, the bubble point is determined by the smallest cross-section of the largest pore. In this case this is the largest pore on the outer shell surface of the fiber. Although hollow fibers may have a similar overall porosity, like Matrimid^®^ fiber *b* and Torlon^®^ fiber *d*, a lower bubble point is expected for the hollow fiber with the larger surface pores, as exhibited in fiber *b*.

### 2.2. Modes of Failure

**Figure 2:**
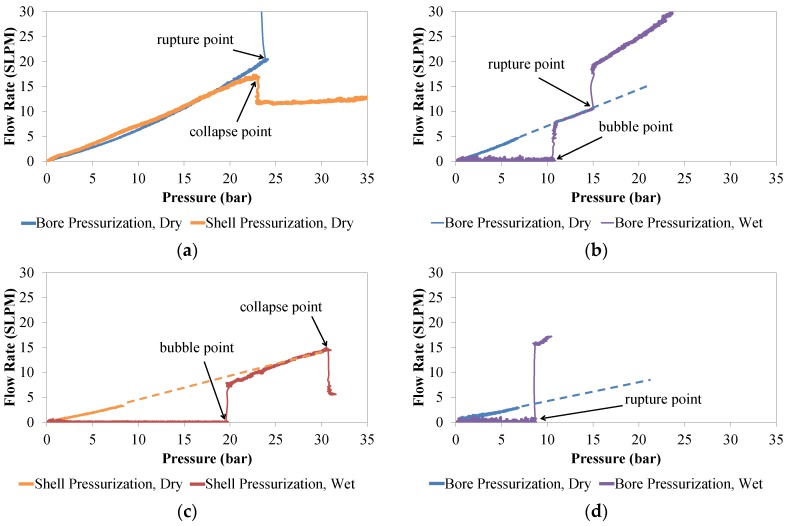
Flow rate (in standard liters per minute, supported ionic liquid membranes (SLPM)) *versus* pressure curves (in bar) depicting: (**a**) rupture/collapse point for dry hollow fibers; (**b**) bubble and rupture points for a wetted hollow fiber pressurized from the bore side; (**c**) bubble and collapse points for a wetted hollow fiber pressurized from the shell side; (**d**) rupture point for a wetted hollow fiber pressurized from the bore side in which the rupture point occurs at a lower pressure than the bubble point. The extrapolated portions of the dry fiber pressurization curves are indicated with a dashed line.

**Figure 3:**
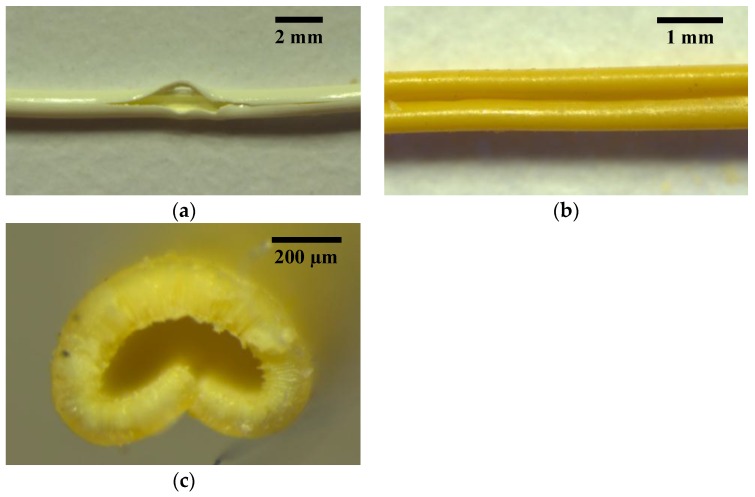
Physical deformations of fiber supports: (**a**) rupture; (**b**) collapse; (**c**) collapse (cross section view).

Hollow fibers were pressure tested from the bore and the shell sides, first dry and then wetted with IL (refer to the Materials and Methods section for further details). Wetted fibers were first tested dry up to 7 bar to provide a baseline response for the hollow fiber support. Fibers exhibited three different failure modes: rupture, collapse, and liquid loss (occurring at the bubble point). These failure modes were determined by comparison of the dry and wet pressurization curves (Figure 2) and by visual inspection at the conclusion of the tests (Figure 3).

Figure 2a depicts both rupture and collapse points in dry hollow fibers that were pressurized from the bore or shell sides, respectively. For both failures, a roughly linear flow rate *versus* pressure curve was observed until failure at ~23 bar. Note that the bore side pressurization curve is slightly concave, due to expansion of the bore and pores under increasing internal pressure, while the shell side pressurization curve is slightly convex due to constriction of the bore under increasing external pressure. For fibers pressurized from the bore side, an abrupt increase in flux indicates that the fiber wall has ruptured (Figure 2a, blue curve). A typical rupture is depicted in Figure 3a, where the fissure occurs along the fiber length due to the high hoop stress associated with a cylindrical pressure vessel [24].

When pressurized from the shell side, an abrupt decrease in flow rate occurs when the fiber collapses and causes a restriction of gas flux through the fiber wall (Figure 2a, orange curve; Figure 3b,c). The buckling of the fiber wall will occur at the weakest point of the hollow fiber cross section [25]. The fiber in Figure 3b collapses along its length; however, collapses did not generally occur throughout the entire length of test specimens, but only in smaller 1–3 cm subsections. In some cases, hollow fibers that are pressurized from the shell side will collapse and ultimately rupture, evidenced by a discontinuity of the fiber wall at the folding point (Figure 3c). A pressurization curve for this case will consist of an abrupt decrease in flow rate after the hollow fiber collapse, followed by an abrupt increase in flow rate after the rupture.

Comparing the pressurization curves for a dry hollow fiber with the same fiber wetted with IL can provide insight into the failure mechanism. Figure 2b shows an example in which an IL wetted hollow fiber is pressurized from the bore side and has zero flow through the fiber wall at low pressures, indicating that all pores are closed off by saturation of the pores with liquid. At a pressure of 11 bar, the bubble point is reached and the flow rate increases abruptly as gas begins to flow convectively through the pores of the membrane. Once all pores are emptied of IL, the pressure curve becomes approximately the same as the previously tested curve for a dry fiber. At a pressure of 15 bar, a second event causes the flow rate to abruptly increase again, at a notably higher rate than for the dry fiber. This indicates that the fiber wall has ruptured.

Figure 2c again shows an IL saturated hollow fiber that is pressurized from the shell side and has zero flow through the fiber wall at low pressures. After the bubble point is reached (~20 bar), the IL empties from the fiber wall pores and the pressure curve roughly overlaps the previously tested curve for the dry fiber. An abrupt reduction in flow rate at a pressure of 30 bar signifies that the fiber wall has collapsed.

In nearly all experiments for this study, wetted hollow fibers exhibited a bubble point before rupturing. However, this trend is not universally true. If a fiber has very small pores but is made from a weak material or contains a manufacturing defect that causes it to be abnormally weakened, then it is also possible for rupture to occur at a lower pressure than the bubble point. In Figure 2d, a wetted fiber has an abrupt increase in flow rate at approximately 9 bar, after which the flow rate is significantly higher than for the dry fiber. The fiber has clearly ruptured without any prior indication of a bubble point, suggesting that the bubble point for this membrane is higher than the rupture pressure. In this case, there is no way to measure the bubble point. However, using the Laplace-Young equation, it may be possible to predict the bubble point if the same type of hollow fiber was pressurized using a different wetting fluid with lower surface tension [9]. This would decrease the bubble point and provide a baseline for predicting the behavior using other liquids with known surface tensions.

### 2.3. Hollow Fiber Pressurization Measurements

**Figure 4:**
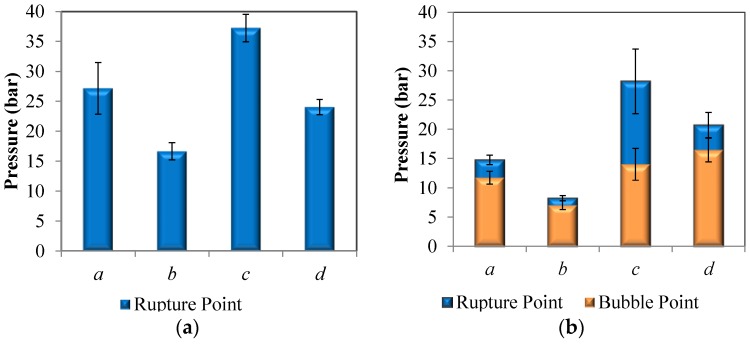

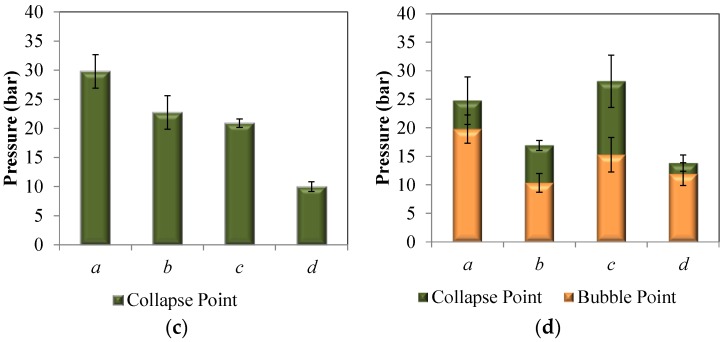
Failure pressures for the four tested hollow fibers: (**a**) pressurized from the bore side, dry; (**b**) pressurized from the bore side, wetted with [C_6_mim][Tf_2_N]; (**c**) pressurized from the shell side, dry; (**d**) pressurized from the shell side, wetted with [C_6_mim][Tf_2_N].

**Figure 5:**
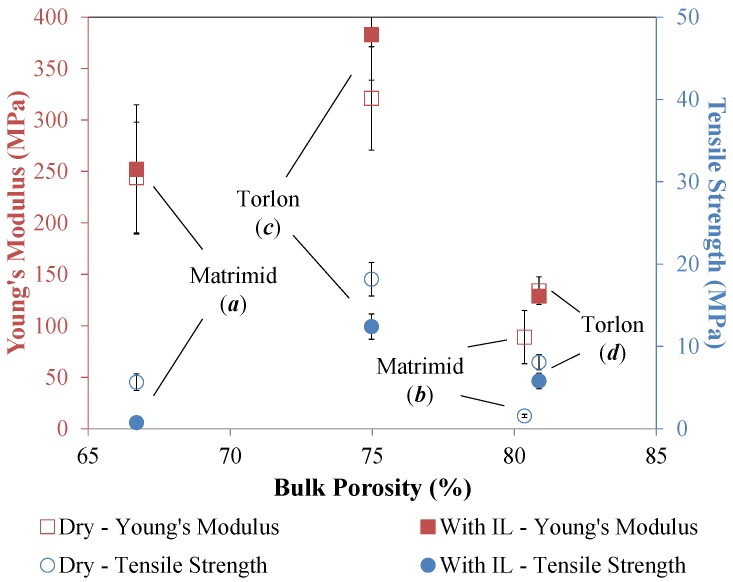
Young’s modulus and ultimate tensile strength of dry hollow fibers and fibers saturated in [C_6_mim][Tf_2_N] at 30 °C.

Failure modes and corresponding pressures were determined for each type of hollow fiber SILM. Fibers pressurized from the bore side exhibited rupture failures when tested dry, shown in Figure 4a. Notably, ruptures occurred at lower pressures for the higher porosity fiber morphologies *b* and *d* due to the reduction in polymer content. Torlon^®^ fibers *c* and *d* exhibited a higher rupture point when pressurized form the bore side than Matrimid^®^ fibers *a* and *b*. The fibers had similar trends in their tensile strength and Young’s modulus, with Torlon^®^ fibers *c* and *d* exhibiting a higher strength and Young’s modulus than Matrimid^®^ fibers *a* and *b* (Figure 5).

Bubble and rupture points for hollow fibers that were wetted with [C_6_mim][Tf_2_N] and pressurized from the bore side are shown in Figure 4b. Neat Matrimid^®^ hollow fibers ruptured at higher pressures than those that were wetted with IL. Torlon^®^ fibers also showed a reduction in rupture pressure when wetted with IL, but to a lesser degree than Matrimid^®^ fibers. This may be due to an interlocking effect that has been predicted for Torlon polymer chains, causing this polymer to resist plasticization [5]. This trend was also observed in the Young’s modulus and tensile strength values for these hollow fibers (Figure 5).

Predictably, bubble points were lower for the high porosity Matrimid^®^ hollow fiber support *b* than low porosity *a*. However, the trend was less straightforward for Torlon^®^ hollow fiber supports *c* and *d*, which had different bulk porosities but similar bubble points. In Figure 4b, the bubble points for *c* and *d* are within the error bars of each other. It should be noted that while the overall porosity of Torlon^®^ hollow fibers *c* is less than *d* (0.75 and 0.81, respectively), that this is not directly related to the bubble point, which is actually a function of the surface pore size. While it is clear in Figure 1 that Matrimid^®^ fiber *b* has a larger surface pore size than *a*, it is not certain that the surface pores of Torlon fiber *d* are larger than *c* since these pores cannot be resolved in the SEM images as shown. Based on the bubble points, the surface pore size is similar for hollow fibers *c* and *d*.

Another interesting trend in Figure 4b is that the rupture points in fibers *a*, *b*, and *d* occurred almost immediately after their bubble points. This is likely not a coincidence, but due to the introduction of convective gas flow through the fiber wall after the bubble point is reached. The abrupt and relatively high rate of gas flow through the fiber pores leads to the deformation of the pore walls, their failure, and ultimately the rupture of the fiber. This trend did not, however, hold true with Torlon fiber c, and was most likely due to the very high tensile strength and Young’s modulus of that fiber (Figure 5). Because this fiber exhibited exceptional strength and stiffness, the pore walls were able to withstand greater convective gas flow before rupture.

Wetted Torlon^®^ fibers exhibited higher rupture and bubble points when fed from the bore side than Matrimid^®^ fibers. When pressurized from the shell side, dry Matrimid^®^ hollow fibers collapsed at approximately the same pressure as the rupture occurs when pressurized from the bore side (Figure 4c). Dry Torlon^®^ hollow fibers withstood less pressure before failure when pressurized from the shell side than from the bore side, and performed slightly worse than comparable Matrimid^®^ hollow fibers under this type of loading. Wetted Matrimid^®^ hollow fibers collapsed at a slightly lower pressure than dry Matrimid^®^ fibers, while wetted Torlon^®^ hollow fibers collapsed at approximately the same pressures as dry Torlon^®^ fibers when experimental error is considered (Figure 4d).

It is noteworthy that wetted Matrimid^®^ fibers were generally more robust when pressurized from the shell side than from the bore side. The collapse pressures for hollow fibers *a* and *b* were higher than their rupture pressures, and bubble points were slightly higher as well (Figure 4d). The increased bubble point for Matrimid^®^ fibers when pressurized from the shell side can be explained as follows. Because Matrimid^®^ hollow fibers have reduced strength under bore side pressurization, they deform more and this causes the pores to stretch, therefore decreasing the bubble point. Conversely, under shell side pressurization the material has increased strength, so the pores undergo less deformation and therefore the bubble point is higher. Wetted Torlon^®^ fibers, on the other hand, had similar collapse and rupture pressures. Likewise, the bubble point for Torlon^®^ fibers was roughly the same whether pressurized from the bore side or shell side when experimental error is considered.

## 3. Materials and Methods 

### 3.1. Materials

All chemicals used in this work were commercially obtained at the highest available purity and were used as received. Huntsman Matrimid^®^ 5218 was from D. H. Litter (Elmsford, NY, USA.) and Torlon^®^ 4000T was from Solvay Advanced Polymers (Alpharetta, GA, USA.). N-methyl-2-pyrrolidone (NMP), methanol, and hexane were from VWR. LiNO_3_ was from Sigma-Aldrich (Saint Louis, MO, USA.). The ionic liquid 1-hexyl-3-methylimidazolium bis(trifluoromethylsulfonyl)imide ([C_6_mim][Tf_2_N]) was from EMD Chemicals (Gibbstown, NJ, USA.). N_2_ gas was ultra-high purity (99.999%) and obtained from the Butler Gas Company (Mckees Rocks, PA, USA.).

### 3.2. Preparation of the Hollow Fibers

Polymer materials were prepared for hollow fiber fabrication as described in detail elsewhere [5]. Briefly, the polymer dopes were prepared by drying the solid polymer materials, dissolving the polymer materials and additives in NMP, and then degassing the mixture. Hollow fibers were fabricated as explained elsewhere using a single-layer spinneret at ambient conditions [26,27]. Briefly, syringe pumps were used to control the core fluid (a mixture of water and NMP, 4:96 wt %) and sheath fluid (polymer solution) flow rates through the spinneret at 60 and 90 mL/h, respectively. The extruded polymer solution fell through a 2 cm air gap into a water quench bath for phase separation, and was subsequently collected onto a winding drum. The fibers were cleaned in solvent and vacuum dried. Single fiber modules were then prepared to facilitate bubble point testing. Modules were prepared by mounting one, 10 cm long hollow fiber in the center of a metal tube using epoxy glue to seal each end. Next, ionic liquid was loaded via pipette into the bore and shell sides of the fiber module and kept at ambient conditions for one week in order to achieve the maximum possible IL loading as previously reported [6].

### 3.3. Bubble Point Measurements

**Figure 6:**
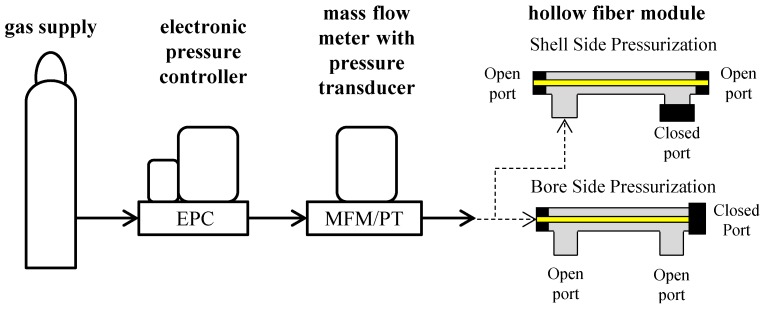
Schematic of bubble point measurement apparatus with bore and shell side pressurization configurations.

Hollow fiber SILMs were subjected to bubble point testing using a custom apparatus (Figure 6). An electronic pressure control valve (GP1, Proportion Air^©^, McCordsville, IN, USA) was used to adjust the pressure of nitrogen gas (N_2_), and a mass flow meter (SLA5860, Brooks^®^ Instruments, Hatfield, PA, USA) was used to monitor flow rate and pressure. Using a LabVIEW^®^ (National Instruments^®^, Austin, TX, USA) computer controlled data acquisition interface, pressure was initially set to zero and gradually increased at a linear rate of 6.9 kPa/s, while recording pressure and flow rate. Measurements were made at ambient temperature (20 °C). Hollow fibers were tested both dry and wetted with IL and were pressurized from either the bore or the shell side (Figure 6). Fibers were pressurized until they either ruptured or collapsed. Before adding ionic liquid to the wetted hollow fiber samples, they were first pressurized dry up to approximately 7 bar. This was used to establish a baseline flow rate-pressure curve for each sample in a relatively low pressure regime in order to avoid damage to the fiber. Then, fibers were saturated with ionic liquid and subsequently re-pressurized until failure. Each experiment was repeated three times. The occurrence of bubble points or ruptures were determined by comparing the dry and wetted flow rate-pressure curves as is explained in the Results and Discussion section.

### 3.4. Hollow Fiber Characterization

The tensile strength and Young’s modulus of the hollow fibers were measured using a dynamic mechanical analyzer (DMA, TA Instruments Q800, New Castle, DE, USA). Tension tests were conducted at a temperature of 30 °C by applying an increasing linear stress to individual fibers at a rate of 0.1 N/min until fracture occurred. Young’s modulus was calculated as the ratio of stress/strain for the first 0.5% strain [24].

Hollow fibers were examined under a Scanning Electron Microscope (SEM, Quanta 600F, Tao Yuan, Taiwan). The SEM samples were prepared by freeze fracturing the hollow fibers in liquid N_2_ and then sputter coating them with Au/Pd 60/40.

The total porosity (or bulk porosity), *v_bulk_*, was determined for each fiber composition through dimensional analysis applying the relationship [15]:

(2) $$\nu_{bulk}=1-\frac{1}{\rho_{poly}}\frac{4m}{\pi \left(OD^{2}-ID^{2}\right)l^{\prime }}\;,$$

where ρ*_poly_* is the density of the polymer, *OD* is the outer diameter of the fiber, *ID* is the inner diameter, *m* is mass, and *l* is length. The density of the neat Matrimid^®^ and Torlon^®^ polymers were measured with a pycnometer (AccuPyc II 1340, Micromeritics, Norcross, GA, USA) to be 1.30 and 1.41 g/cm^3^, respectively.

## 4. Conclusions

The bubble point method was used to determine the maximum trans-membrane pressures of hollow fiber supported ionic liquid membranes. Bubble points and rupture/collapse pressure were reported for Matrimid^®^ and Torlon^®^ hollow fibers of various porosity levels. When pressurized from the bore side, Torlon^®^ hollow fibers exhibited higher rupture pressures than Matrimid^®^ fibers, and were less affected by plasticization due to IL exposure. However, dry Matrimid^®^ hollow fibers had higher collapse pressures than Torlon^®^ when pressurized from the shell side, while wetted Matrimid^®^ and Torlon^®^ fibers had similar collapse pressures. Bubble points were generally similar for SILMs whether they were fed from the bore or the shell sides, although slightly higher for Matrimid^®^ fibers that were pressurized from the shell side rather than the bore side. Bubble points were inversely proportional to the size of the outer surface pores. Tensile tests showed a reduction in tensile strength and Young’s modulus with increasing porosity for both hollow fiber support materials, which was mirrored by the measured rupture pressures. Although the results from this study show that the bubble point can be reasonably high for these membranes, it would be beneficial to further study the long term stability of the membranes at various transmembrane pressures. This would provide insight into the maximum operating pressure that can be sustained over the long term without degrading the gas transport properties of the membrane.

## Figures and Tables

**Table 1:** Composition of the polymer dope.

Membrane	Matrimid^®^ (wt %)	Torlon^®^ (wt %)	NMP (wt %)	LiNO_3_ (wt %)
*a*	21	0	75	4
*b*	18	0	78	4
*c*	0	18	78	4
*d*	0	14	82	4

**Table 2:** Dimensions and porosity of the hollow fibers.

Property	*a*	*b*	*c*	*d*
*OD* (μm)	563 ± 13	639 ± 57	545 ± 12	590 ± 17
*ID* (μm)	332 ± 49	357 ± 24	289 ± 18	335 ± 10
*v_bulk_*	0.67 ± 0.04	0.79 ± 0.04	0.75 ± 0.03	0.81 ± 0.04

## Abbreviations

The following abbreviations are used in this manuscript:

MDPI: Multidisciplinary Digital Publishing Institute
DOAJ: Directory of open access journals
SILM: Supported ionic liquid membrane
IL: Ionic liquid
[C_6_mim][Tf_2_N]: 1-hexyl-3-methylimidalzolium bis(trifluoromethylsulfonyl)imide
[C_4_mim][EtSO_4_]: 1-ethyl-3-methylimidazolium ethyl sulfate
[C_3_NH_2_mim][Tf_2_N]: N-aminopropyl-3-methylimidazolium bis(trifluoromethylsulfonyl)imide
NMP: N-methyl-2-pyrrolidone
